# A randomized trial of preoperative oral carbohydrates in abdominal surgery

**DOI:** 10.1186/1471-2253-14-93

**Published:** 2014-10-17

**Authors:** Fatos Sada, Avdyl Krasniqi, Astrit Hamza, Agreta Gecaj-Gashi, Besnik Bicaj, Floren Kavaja

**Affiliations:** Clinic of Anesthesiology and Intensive Care, University Clinical Center of Kosovo, Rr. Hyzri Talla, hy 7/8, Bregu i Diellit, Zona e Lindjes, Prishtina, Kosovo; Clinic of Surgery, University Clinical Center of Kosovo, Prishtina, Kosovo

**Keywords:** Carbohydrates, Fasting, Thirst, Preoperative use of carbohydrates, Postoperative period

## Abstract

**Background:**

Carbohydrate-rich liquid drinks (CRLDs) have been recommended to attenuate insulin resistance by shortening the preoperative fasting interval. The aim of our study the effect of preoperative oral administration of CRLDs on the well-being and clinical status of patients.

**Methods:**

A randomized, double blind, prospective study of patients undergoing open colorectal operations (CR) and open cholecyctectomy (CH) was conducted. Patients were divided into three groups: study, placebo, and control. Visual analogue scale (VAS) scores for seven parameters (thirst, hunger, anxiety, mouth dryness, nausea, weakness and sleep quality) were recorded and compared for two different time periods (up to 24 h postoperatively and from 36 to 48 h postoperatively). The Simplified Acute Physiology Score changes (SAPS)-II between the three groups were also studied.

**Results:**

There were 142 patients American Society of Anesthesiology (ASA) I or II enrolled in the study (CR = 71 and CH = 71). There were no significant differences in postoperative SAPS-II scores or lengths of hospital stay (LOS) between the groups. However, in CR patients, the degree of thirst was partially improved by drinking CRLDs (P = 0.027). In CH patients, on the other hand, feelings of thirst, hunger, mouth dryness, nausea and weakness showed significant improvement (P < 0.05).

**Conclusion:**

Oral administration of carbohydrate-rich liquid drinks (CRLDs) improves the well-being in patients undergoing CH, but the effect is less evident in patients undergoing CR. No significant improvements were seen in clinical status or in length of hospital stay in either group.

**Trial registration:**

ANZCTR.org.au: ACTRN12614000995673 (registered on 16/09/2014).

**Electronic supplementary material:**

The online version of this article (doi:10.1186/1471-2253-14-93) contains supplementary material, which is available to authorized users.

## Background

Insulin resistance, as a stress inducer, is a positive protective reaction against surgery [1]. As a response to injury (surgery), activation of neuroendocrine and inflammation systems occurs as a protective reaction that initiates insulin resistance [2, 3]. However, beyond a point, this resistance begins to have negative consequences for patient health [4, 5]. One of the mechanisms to attenuate insulin resistance is preoperative administration of carbohydrate-rich liquid drinks (CRLDs) [6].

The more complex or lengthy the surgery, (i.e., the severity: tissue trauma, blood loss), the more severe the development of insulin resistance [7]. In cholecystectomy procedures, insulin sensitivity was reduced by 56% in one study (P < 0.01) [8] while in major colorectal surgery interventions, insulin sensitivity can be reduced as much as 90% [1].

The nothing by mouth rule (NPO) from the night before surgery, which extends throughout the postoperative period (can last 12–16 h), in recent years, has been under scrutiny [9–11]. Studies have revealed that fasting can intensify perioperative insulin resistance [12, 13]. Administration of an iso-osmolar, carbohydrate rich, beverage preoperatively is the most efficient and natural way to provide certain quantities of carbohydrates and does not pose any threat from vomiting or aspiration if taken no sooner than 2 h before anesthesia induction [14–16]. As a result of these studies, CRLDs are recommended to minimize preoperative fasting [17, 18].

It has been reported also that preoperative consumption of CRLDs reduces patient discomfort and anxiety in the perioperative period, [19] and resulted in a shorter hospital stay [20]. In many countries, preoperative administration of carbohydrates is included in the institutional protocols [21–23], although this is not wide spread practice. These recommendations rely on studies that have suggested that administration of carbohydrates reduces metabolic stress, insulin resistance and attenuates the nitrogen losses, [24] as well as improving muscular strength in larger groups [25].

We designed the study to assess whether preoperative oral administration of CRLDs has an impact on patient well-being, clinical status, time to regain activities of daily living, and reduction in the length of hospital stay, in patients undergoing open abdominal surgery (CR, CH).

## Methods

This prospective, double-blind, randomized trial was performed between January 2010–January 2012 at the University Clinical Center of Kosovo. Patients were randomized into one of three groups: a study group, placebo, and control. In this study, the placebo beverage was indistinguishable in appearance and taste as possible from the special formula. For this purpose, a colleague blinded to the rest of the study (patients, clinical and biochemical parameters recorded during the study) was asked to prepare and label beverages according to the randomization codes. All other researchers were blinded to those codes. The study group received 800 mL (per os) of carbohydrate beverage in the evening before surgery (22:00) and an additional 400 mL 2 h before anesthesia induction. The placebo group received a non-caloric colorless liquid with the same taste and without carbohydrates in the same amount as the patients in the study group. The control group did not receive any of these drinks and were subject to the traditional preoperative fasting. Each procedure was performed following the written consent obtained from each patient. Inclusion criteria were that patients were older than 18 years, undergoing an operation of the colon and rectum for benign and malignant diseases, or open abdominal cholecystectomy for chronic cholecystitis. Each group of patient was analyzed and reported separately. Exclusion criteria were type 1 or 2 diabetes mellitus, stomach emptying disorders or documented gastric esophageal reflex disease, emergency surgery interventions, or refusal of the patient to participate in the trial. The Ethics Committee of the Medical Faculty/ University of Pristina approved the trial (protocol number: 2128, date 31.05.2010).

All beverages were packed into identical bottles marked with numbers (containing carbohydrates or placebo) by an assigned person who did not provide the liquids to the patients and did not take part in the evaluation of the results. For the study group, the beverage contained 12.5% carbohydrates, 50 kcal/100 mL, 285 mOsmol/kg (NutriciapreOp, Nutricia Ltd., Wiltshire, UK, who provided the supplements.). Patients were premedicated in the evening before the surgery with 5 mg intramuscular diazepam and 0.3 ml subcutaneous Fraxiparine (Nadoparin, GlaxoSmithKline, UK). Patients were asked to have a regular diet for at least two days prior to admission to the clinical ward. To achieve standardization across the participating departments, patients had a bowel preparation with a 2 × 45 ml dose of sodium phosphate given orally the day before surgery (at 1500 and 2000). Intraoperative fluid management was depended on the anesthetist’s preference. The patient was administered prophylactic antibiotics 30 to 60 minute before the first incision (A first or second generation cephalosporin plus metranidazol given by i.v. route, single dose of each). No IV fluids were given to any of the groups preoperatively. Anesthesia induction was performed with intravenous midazolam 2 mg, fentanyl (3–4 mcg/kg) and thiopental (4–6 mg/kg). Pancuronium (0.1 mg/kg) or atracurium (0.5 mg/kg) was used for muscular relaxation. General anesthesia was maintained with continual propofol and intermittent bolus doses of fentanyl. Postoperative pain was treated with NSAID and tramadol hydrochloride, while for PONV there was not used any prophylactic drug.

### Endpoints

#### Primary endpoints

##### Patient well-being

Thirst, anxiety, hunger, mouth dryness, nausea, weakness and sleep quality were assessed using visual analogue scale (VAS) scores 1–10. [26] In order to have a clearer picture if CHO drinks do or not have an impact in postoperative well being of the patients VAS score questionnaire was filled twice, within 24 h following the surgery (in the morning of the first postoperative day) and between 36 to 48 h following the surgery (in the morning of the second postoperative day).

##### Patient clinical status

Clinical evaluation by the Simplified Acute Physiology Score (SAPS-II), [27] was performed within 24 h following the surgery. Lowest or highest recorded BP and heart frequency following 24 h after surgery was used to calculate SAPS II score.

##### Length of hospital stay

The length of hospital was recorded for each patient.

#### Secondary endpoint

##### Convalescence

Regaining daily activities.

Data are presented using tabular presentations. Data processing was performed using the statistical package InStat 3 (San Diego, California, USA). The following statistics were calculated: arithmetic mean, standard deviation, and standard error of the mean. Minimal and maximal values were also recorded. For statistical testing, we used one-way analysis of variance (ANOVA) and the Bonferroni test for multiple comparisons for parametric data and the Kruskal-Wallis (KW) test and the Dunn test for multiple comparisons for non-parametric data. Differences were significant for values of P < 0.05.

## Results

There were 162 patients enrolled in the study, form which 142 were analyzed (CR = 71 and CH = 71) (see Additional file 1 - CONSORT diagram). All patients were American Society of Anesthesiology (ASA) I or II. Characteristics of the study, placebo and control groups are presented for CR and CH patients in Tables 1 and 2. No statistically significant differences were found in mean ages between the three groups in CR patients (F = 0.54, P = 0539, P > 0.05). No statistically significant differences in the mean duration of surgery between the CR study, placebo and control groups (KW = 0.96, P >0.05). Also, between the groups of CH patients no difference was noted for demographic data (age F = 0.75, P > 0.05). The results of VAS score comparisons for seven parameters, 0–24 h and 36–48 h postoperatively, for both the CR and CH groups are presented in Table 3.

**Table 1:** **Demographic and other clinical data for colorectal surgery patients- gender, age and surgery duration were identical among the three study groups**

	Study	Placebo	Control
**Gender**			
n (%)	22 (100)	23 (100)	26 (100)
M	11 (50)	9 (31.1)	13 (50)
F	11 (50)	14 (60.9)	13 (50)
**Age (years)**			
Mean	57.9± 12.0	56.6± 13.0	53.8± 15.8
**Surgery duration (hours)**			
Mean	3.3± 0.5	3.1± 0.4	3.1± 0.5

**Table 2:** **Demographic and other clinical data for cholecystectomy patients- gender, age and surgery duration were identical among the three study groups**

	Study	Placebo	Control
**Gender**			
n (%)	22 (100)	23 (100)	26 (100)
M	6 (27)	6 (26)	8 (31)
F	16 (73)	17 (74)	18 (69)
**Age (years)**			
Mean	55.8± 13.5	54.8± 15.1	59.1± 12.0
**Surgery duration (hours)**			
Mean	1.2 ± 0.4	1.2± 0.3	1.3± 0.4

**Table 3:** **Well-being by VAS score**

		Colorectal patients						Cholecystectomy patients					
		N S=22; P=23; C=24						N S=22; P=23; C=24					
**VAS score**		0-24h, postop.			36-48h, postop.			0-24h, postop.			36-48h, postop.		
		S	P	C	S	P	C	S	P	C	S	P	C
**Thirst**	Median	3	2	4	2	1	2	3	2	4	2	1	2
	Min	1	1	1	1	1	1	1	1	1	1	1	1
	Max	5	5	7	3	3	5	5	5	7	3	3	5
		Gr. S vs. Gr. P; P>0.05			Gr. S vs. Gr. P; P>0.05			Gr. S vs. Gr. P; P>0.05			Gr. S vs. Gr. P; P>0.05		
		Gr. S vs. Gr. C; P>0.05			**Gr. S vs. Gr. C; P<0.05**			**Gr. S vs. Gr. C; P<0.05**			Gr. S vs. Gr. C; P>0.05		
		**Gr. P vs. Gr. C; P<0.05**			Gr. P vs. Gr. C; P>0.05			**Gr. P vs. Gr. C; P<0.05**			**Gr. P vs. Gr. C; P<0.05**		
**Hunger**	Median	3	3	4	2	2	3	3	2	4	2	2	3
	Min	1	1	1	1	1	1	1	1	1	1	1	1
	Max	5	5	8	4	4	6	5	5	8	4	4	6
		Gr. S vs. Gr. P; P>0.05			Gr. S vs. Gr. P; P>0.05			Gr. S vs. Gr. P; P>0.05			Gr. S vs. Gr. P; P>0.05		
		Gr. S vs. Gr. C; P>0.05			Gr. S vs. Gr. C; P>0.05			**Gr. S vs. Gr. C; P<0.05**			Gr. S vs. Gr. C; P>0.05		
		Gr. P vs. Gr. C; P>0.05			Gr. P vs. Gr. C; P>0.05			**Gr. P vs. Gr. C; P<0.05**			Gr. P vs. Gr. C; P>0.05		
**Anxiety**	Median	3	1	2	1	1	1.5	2	1	2	1	1	1.5
	Min	1	1	1	1	1	1	1	1	1	1	1	1
	Max	3	4	6	3	3	5	3	4	6	3	3	5
		Gr. S vs. Gr. P; P>0.05			Gr. S vs. Gr. P; P>0.05			Gr. S vs. Gr. P; P>0.05			Gr. S vs. Gr. P; P>0.05		
		Gr. S vs. Gr. C; P>0.05			Gr. S vs. Gr. C; P>0.05			Gr. S vs. Gr. C; P>0.05			Gr. S vs. Gr. C; P>0.05		
		Gr. P vs. Gr. C; P>0.05			Gr. P vs. Gr. C; P>0.05			Gr. P vs. Gr. C; P>0.05			Gr. P vs. Gr. C; P>0.05		
**Mouth dryness**	Median	5	4	6	2	2	3	5	4	6	2	2	3
	Min	3	3	3	1	1	1	3	3	3	1	1	1
	Max	7	7	9	4	4	5	7	7	9	4	4	5
		Gr. S vs. Gr. P; P>0.05			Gr. S vs. Gr. P; P>0.05			Gr. S vs. Gr. P; P>0.05			Gr. S vs. Gr. P; P>0.05		
		Gr. S vs. Gr. C; P>0.05			Gr. S vs. Gr. C; P<0.05			**Gr. S vs. Gr. C; P<0.05**			**Gr. S vs. Gr. C; P<0.05**		
		Gr. P vs. Gr. C; P>0.05			Gr. P vs. Gr. C; P>0.05			**Gr. P vs. Gr. C; P<0.05**			**Gr. P vs. Gr. C; P<0.05**		
**Nausea**	Median	1	3	2.5	1	2	2	1	3	2.5	1	2	2
	Min	1	1	1	1	1	1	1	1	1	1	1	1
	Max	5	6	6	3	3	5	5	6	6	3	3	5
		Gr. S vs. Gr. P; P>0.05			Gr. S vs. Gr. P; P>0.05			Gr. S vs. Gr. P; P>0.05			Gr. S vs. Gr. P; P>0.05		
		Gr. S vs. Gr. C; P>0.05			Gr. S vs. Gr. C; P<0.05			**Gr. S vs. Gr. C; P<0.05**			Gr. S vs. Gr. C; P>0.05		
		Gr. P vs. Gr. C; P>0.05			Gr. P vs. Gr. C; P>0.05			Gr. P vs. Gr. C; P>0.05			Gr. P vs. Gr. C; P>0.05		
**Weakness**	Median	4	3	3	1	2	3	4	3	3	2	2	3
	Min	1	1	1	1	1	2	1	1	1	1	1	1
	Max	6	6	6	3	4	4	6	6	6	5	4	4
		Gr. S vs. Gr. P; P>0.05			Gr. S vs. Gr. P; P>0.05			Gr. S vs. Gr. P; P>0.05			Gr. S vs. Gr. P; P>0.05		
		Gr. S vs. Gr. C; P<0.05			Gr. S vs. Gr. C; P>0.05			**Gr. S vs. Gr. C; P<0.05**			Gr. S vs. Gr. C; P>0.05		
		Gr. P vs. Gr. C; P>0.05			Gr. P vs. Gr. C; P>0.05			Gr. P vs. Gr. C; P>0.05			Gr. P vs. Gr. C; P>0.05		
**Sleep quality**	Median	3	3	3.5	2	3	3	3	3	3.5	2	3	3
	Min	1	1	1	1	1	1	1	1	1	1	1	1
	Max	5	7	6	5	5	6	5	7	6	5	5	6
		Gr. S vs. Gr. P; P>0.05			Gr. S vs. Gr. P; P>0.05			Gr. S vs. Gr. P; P>0.05			Gr. S vs. Gr. P; P>0.05		
		Gr. S vs. Gr. C; P>0.05			Gr. S vs. Gr. C; P>0.05			Gr. S vs. Gr. C; P>0.05			Gr. S vs. Gr. C; P>0.05		
		Gr. P vs. Gr. C; P>0.05			Gr. P vs. Gr. C; P>0.05			Gr. P vs. Gr. C; P>0.05			Gr. P vs. Gr. C; P>0.05		

For each evaluation statistic, the bold numbers denotes significant statistical difference.

### Colorectal group

For the six out of seven parameters of VAS scores (hunger, anxiety, mouth dryness, nausea, fatigue and sleep quality), we found no statistically significant differences between the groups, 0–24 h and 36–48 h after CR surgery (P > 0.05). Multiple comparisons gave the same results for these parameters (study group vs. placebo group, P > 0.05; study group vs. control group, P > 0.05; placebo group vs. control group, P > 0.05).

During the first 24 h following the surgery, thirst was the only parameter statistically significant different between the placebo and control groups (KW = 7.19, P = 0027, i.e. P < 0.05). During postoperative time interval between 36–48 h, the only significant difference in thirst was between the study versus control group (Table 3). However, no significant statistical difference between the mean values of SAPS scores were found (KW = 0.98, P > 0.05) (Table 4). Multiple comparisons gave the same results (study group vs. placebo group, P > 0.05; study group vs. control group, P > 0.05; placebo group vs. control group, P > 0.05).

**Table 4:** **The parameters of SAPS II score values by group in colorectal patients- clinical evaluation by SAPS-II was performed within 24 h following the surgery**

SAPS II score	Study Gr.	Placebo Gr.	Control Gr.
Number of patients	22	23	23
Mean	13.0	12.1	11.0
SD	6.5	6.6	7.8
SEM	1.39	1.37	1.62
Min	0	0	0
Max	29	23	32
Kruskal Wallis test	KW=0.98, P>0.05		
Dunn Multiple Comparisons Test	S. Gr. vs. P. Gr.; P>0.05		
	S. Gr. vs. C. Gr.; P>0.05		
	P. Gr. vs. C. Gr.; P>0.05		

Gr. S: study group, Gr. P: placebo group, Gr. C: control group.

LOS for the study group was 10.0 ± 3.4 days, control group 10.1 ± 4.7 days, and placebo group 10.3 ± 2.7. There was no significant statistical difference in the LOS between the groups (KW = 1.67, P > 0.05). We found no statistically significant differences in the time to return of bowel sounds, t flatus, defecation, sitting out of bed, oral intake, walk with assistance, walk without assistance, or nasogastric drainage for the patients between the groups (KW, P > 0.05), (study group vs. placebo group, P > 0.05; study group vs. control group, P > 0.05; placebo group vs control group, P > 0.05).

### Cholecystectomy group (CH)

There were statistically significant differences (P < 0.05) in the degree of thirst, hunger, mouth dryness, nausea and weakness in the study group compared with the control group in less than 24 h following the surgery, while the differences in degree of anxiety and sleep quality were not significant (P > 0.05) (Table 3). However, 36–48 h following surgery, only mouth dryness was found to be significant, when comparing study group vs. control group (P < 0.05).The degree of anxiety, nausea, weakness and sleep quality were not significant (P > 0.05).

Comparing study group vs. placebo group, in two time intervals, lees then 24 h and 36–48 h there were no significant differences in any of the seven well being parameters.

LOS for the study group was 4.4 ± 0.8 days, control group 4.4 ± 1.0 days, and placebo group 4.6 ± 1.1. There was no significant statistical difference in the LOS between the groups (KW = 2.0, P > 0.05). In addition, the SAPS II score and recovery time were not statistically different between the groups (study group vs. placebo group; P > 0.05, study group vs. control group; P > 0.05, placebo group vs. control group; P > 0.05).

## Discussion

We have attempted to answer the question if the postoperative well-being of the patients is improved by the preoperative administration of CRLDs. While, thirst was the only parameter that was improved in patients undergoing colorectal surgery, all other well-being parameters of VAS score were not affected. However, in patients undergoing open cholecystectomy, our data reveal that the postoperative well-being of patients who received CRLDs in the preoperative period improved five out of seven well-being parameters (thirst, hunger, mouth dryness, nausea and weakness) when compared the study group vs. control group, or for three parameters when compared the placebo group vs. control group (P < 0.05).

Attenuated thirst and a reduced need for perioperative administration of inotropic drugs when taking CRLDs have been observed in patients undergoing open heart surgery interventions [28]. Hausel and colleagues, [14] utilizing VAS for patients categorized under ASA I–II undergoing abdominal surgery (n = 252), found no differences between the groups (CHO and placebo) in the degree of thirst, although there was a significant difference compared with the control group. However, in the same study it was reported that patients who received CRLDs experienced a significant reduction in anxiety and hunger.

In our study there was no difference in SAPS II score or length of hospital stay, and other clinical data were the same for both patients undergoing colorectal and cholecystectomy operations. In contrast to the results of this study, De Aguilar-Nascimento and colleagues conducted a study of 60 female patients undergoing cholecystectomy, patients with carbohydrates intake had reduced gastrointestinal discomfort (vomiting and abdominal distension), as well as a shorter hospital stay [29]. Furthermore, intake of these drinks, which reduces preoperative fasting, demonstrated positive effects on thirst, hunger, perioperative anxiety and muscular strength [29, 24]. On the other hand, Henriksen and colleagues reported results from a small study (n = 48) prior to elective bowel resection, in which their patients showed no differences, even for thirst [27]. In contrast our data, as reported elsewhere [30], show that thirst, as a main component in preoperative discomfort, is effectively reduced by the intake of clear liquids. In addition, in minor surgeries such is laparoscopic cholecystectomy, another study showed no differences in postoperative sleep or well-being, [31] while yet another study indicated less nausea and vomiting for the group with carbohydrate intake compared with fasting patients [32].

An important question arises from our study: why did the study group have greater improvement in patients undergoing cholecystectomy than those undergoing colorectal surgical interventions when taking CRLDs? Results varied between different patients population, investigating the same parameters. In both/different types of surgical operations on patients with varying postoperative insulin resistance the results have been inconsistent. In colorectal operations, which result in more insulin resistance, there were no significantly different results compared with cholecystectomy operations where well-being parameters were statistically different in a positive way for patients drinking a carbohydrate versus control group. Reason might be longer duration of operation in colorectal group where there was enough time to replace volume deficit that exists maybe in control group, so in postoperative period both groups were equally balanced with fluids. This didn’t happen in cholecystectomy operations due to shorter length of operation. This is only an assumption since measuring IV volume loading during operation was not a part of this study. In this study, also majority of results when comparing study group vs. placebo group do not show any significant difference. There are other studies that do have similar results such as Mathur et al. were CHO treatment did not improve postoperative fatigue or length of hospital stay after major abdominal surgery, [33] or Hausael at al. found no differences between the groups (CHO and placebo) in the degree of thirst [14]. This might bring us to hypothesis that volume of oral fluids taken is more important than the energy that they posses based on carbohydrate concentration.

### Study limitations

This study has its own limitations. It is limited by small sample size e.g. small number of patients per group. Approval from Ethical Committee for the study to last two years, limited the number of patients included in research.

Although previous authors [1, 7, 8] talk about difference in insulin resistance in colorectal operations and in cholecystectomy operation, in our study we did not measure insulin resistance or insulin levels since it was not our aim. We measured glucose levels in blood pre and postoperatively as part of routine lab for this project.

## Conclusions

Preoperative oral intake of carbohydrates yields different postoperative well-being (VAS) scores for patients undergoing different operations. The only positive effect on patients undergoing colorectal operations was an attenuation of thirst (36–48 h postoperatively). For patients undergoing cholecystectomy operations, the VAS score was significantly better in patients taking carbohydrate drinks for thirst, hunger, mouth dryness, nausea and weakness. Other aspects studied showed no significant differences among the study group, control group or the placebo group regardless of the type of operation, e.g. clinical status (SAPS-II score), patient convalescence and the length of hospital stay.

## Author’s information

FS is a specialist of Anesthesiology and Intensive Care and works at the University Clinical Center of Kosovo. FS is an Assistant Professor in Department of Anesthesiology and Surgery on Faculty of Medicine at the University of Pristina. He holds an MS degree, and is now working on a PhD doing clinical research on the benefits of preoperative usage of carbohydrate reach liquid drinks.

## Electronic supplementary material

Additional file 1: **CONSORT Flow Diagram.** Flow diagram of subject progress through the phase of a randomized trial. (DOC 34 KB)
