# Supplementary material for: A randomized trial of preoperative oral carbohydrates in abdominal surgery
Source: BMC Anesthesiol. 2014 Oct 17;14:93. doi: 10.1186/1471-2253-14-93 (PMC4216365; doi:10.1186/1471-2253-14-93)
Supplement: Supplementary file 1 — Additional file 1: CONSORT Flow Diagram. Flow diagram of subject progress through the phase of a randomized trial. (DOC 34 KB) [file 12871_2013_307_MOESM1_ESM.doc]

**CONSORT Flow Diagram**

**Allocation**

**Enrollment**

**Analysis**

**Follow-up**

Assessed for eligibility n=162

Excluded n= 17

♦  Not meeting inclusion criteria n=16

♦  Declined to participate n= 1

Allocated to intervention n= 45

Received intervention n= 45

Randomized n= 145

Allocated to intervention n= 46

Received intervention n= 46

Allocated to intervention n= 54

Received intervention n= 54

Analysed n=46
♦ Excluded from analysis n= 2

Lost to follow-up n= 0

Analysed n=52
♦ Excluded from analysis n=1

Lost to follow-up n= 1

Analysed n=44
♦ Excluded from analysis n= 1

Lost to follow-up n= 0

Control

Study

Placebo

**Flow diagram of subject progress through the phase of a randomized trial**
